# The Efficacy of Palmitoylethanolamide (Levagen+) on the Incidence and Symptoms of Upper Respiratory Tract Infection—A Double Blind, Randomised, Placebo-Controlled Trial

**DOI:** 10.3390/nu15204453

**Published:** 2023-10-20

**Authors:** Amanda Rao, Rachael Skinner, David Briskey

**Affiliations:** 1 RDC Clinical, Level 3/252 St. Pauls Terrace, Brisbane 4006, Australia; rachael@rdcglobal.com.au (R.S.); d.briskey@uq.edu.au (D.B.); 2 School of Medicine, University of Sydney, Sydney 2006, Australia; 3 School of Human Movement and Nutrition Sciences, University of Queensland, Brisbane 4072, Australia

**Keywords:** palmitoylethanolamide, URTI, cold, flu

## Abstract

Introduction: Upper respiratory tract infections (URTIs) are caused by bacteria or viruses, with the most common causes being the common cold and influenza. The high occurrence of URTI means therapies that are effective with minimal side effects are in constant demand. Palmitoylethanolamide (PEA) is a signaling lipid previously shown to be effective in improving the incidence of URTIs. The aim of this study was to assess the effectiveness of PEA (Levagen+) on URTI incidence, duration, and severity. Methods: Participants (*n* = 426) consumed either 300 mg of Levagen+ or a placebo (maltodextrin) twice daily for 12 weeks. Participants completed the Wisconsin Upper Respiratory Symptom Survey 24 questionnaire daily upon the commencement of symptoms until symptoms subsided. Results: The Levagen+ group reported fewer URTI episodes (39 vs. 64) compared to the placebo group. The Levagen+ group reported a significant reduction in the median severity score of URTI symptoms for scratchy throat (3 vs. 7) and cough (2 vs. 7) compared to the placebo group. Conclusions: The results of this study show Levagen+ to be safe and effective in reducing the incidence and symptoms associated with URTIs.

## 1. Introduction

Upper respiratory tract infections (URTIs) are caused by an infection of the mucosal lining of the upper airway. URTI symptoms include coughing, sneezing, stuffy or runny nose, fever, and scratchy or sore throat [1,2]. Sources of infection typically originate from either bacteria or viruses [3], with the most common causes being the common cold and influenza [4]. On average, adults have 2–4 episodes of the common cold per year and children have between 6 and 10 episodes [5]. Due to the frequency of occurrence, URTIs require prophylactic and/or treatment options for symptoms that have minimal to no side effects.

Palmitoylethanolamide (PEA) is an endocannabinoid-like bioactive signaling lipid that is part of the *N*-acylethanolamine (NAE) family [6,7]. In the case of cold and flu infections, where there is an increase in inflammatory cytokine production, PEA is proposed to work to modulate interleukins and downregulate mast cell production at inflammation sites [8]. PEA initiates NF-κB pathways via the activation of PPAR receptors, with a high affinity for PPAR-α, and works in a concentration-dependent manner to decrease NLRP3 and inflammasome activation [9]. The anti-inflammatory effects of PEA allow it to reduce the expression of cytokines released from macrophages [8]. Overall, the known mechanisms of action of PEA support the observation that it is able to decrease the symptoms associated with URTIs.

To date, most literature stating the efficacy of PEA on cold and flu symptoms is based on theoretical evidence or animal studies, rather than human clinical studies. Previous human clinical studies have indicated that PEA is an effective treatment in reducing cold and flu symptoms [10,11], but these studies are almost 50 years old. Studies published by Masek and colleagues (1974) found subjects supplemented daily with 1800 mg of PEA for 12 days showed a reduction in episodes of fever, sore throat, and headaches compared to placebo groups [10]. Masek and colleagues also showed that PEA prophylactic supplementation for 8 weeks resulted in a decrease in the incidence of cold and flu from 40% to 32% [10]. A study conducted by Plesnik and colleagues (1977) showed children supplemented daily with 600 mg of PEA had a lower occurrence of acute respiratory tract infections compared with a placebo [11]. Therefore, there is a need for new studies to investigate the effects of modern PEA formulations on cold and flu symptoms.

Recent human clinical studies have focused more on the potential effects of PEA on inflammation associated with COVID-19. PEA has been shown to be effective against respiratory symptoms caused by increased inflammation associated with COVID-19 [12]. Albanese and colleagues (2022) showed that supplementation with ultramicronized PEA reduced markers of inflammation (CRP, IL-6, and neutrophils to lymphocytes ratio) while the placebo group exhibited an increase in oxidative markers (free oxygen radicals test) [13]. A study conducted by Fonnesu and colleagues (2022) showed that PEA could bind to the SARS-CoV-2 protein, and this causes a decrease in viral infection by approximately 70% [14]. PEA was also shown to dismantle lipid droplets, which prevented SARS-CoV-2 from utilising the droplets for energy and protection against immune responses [14]. Fessler and colleagues (2022) studied the effectiveness of 600 mg of PEA twice daily on proinflammatory markers in COVID-19 patients. Fessler and colleagues showed that supplementation with PEA significantly decreased sP-selectin, IL-1β, and IL-2 markers [15]. The sP-selectin marker is essential for clearing infectious agents and foreign particles, as well as the propagation of inflammatory responses [15]. Therefore, with a reduction in sP-selectin and pro-inflammatory cytokines IL-1β and IL-2, the results of the study by Fessler and colleagues suggest that PEA may reduce the appearance of infectious agents and foreign particles and the resulting inflammation, thereby preventing the need for the body’s defense response to be initiated.

However, it is important to note that both endogenous levels of PEA and exogenous PEA administration have previously been reported to be insufficient in mitigating a significant clinical response due to poor absorption, resulting in low plasma concentrations [16,17]. When PEA is combined with dispersion technology (i.e., Levagen+), PEA absorption is significantly increased, leading to higher plasma concentration levels that may enable a therapeutic effect [18]. To the best of our knowledge, apart from some historical evidence for the use of PEA in cold and flu symptoms, no recent studies on PEA and cold and flu symptoms have been published. Therefore, further research is needed to establish its effectiveness and safety with respect to applications on cold and flu symptoms. The results from studies to date suggest that PEA has positive treatment effects and can be effectively used as a prophylactic for URTI symptoms. However, due to the age of some of the studies and the seeming lack of studies in recent decades, additional studies are required to establish the effects of new PEA formulations. The aim of the current study was to explore the efficacy of Levagen+ on the incidence, severity, and duration of URTIs compared to a placebo in otherwise healthy adults. It was hypothesised that those supplemented with Levagen+ would have a reduction in the incidence, severity, and duration of URTIs compared to the placebo.

## 2. Methods

This was a double-blind, randomised, placebo-controlled trial conducted over 12 weeks that utilised an active group (Levagen+) and a placebo group (maltodextrin). Potential participants were provided with the participant information sheet, and following initial screening via a telehealth consultation, acceptable participants gave their written consent to participate in the study and completed baseline measures. This trial was registered with ANZTCR: number ACTRN12620000846921.

Four hundred and twenty-six participants aged between 18 and 65 years old were recruited from databases and public media outlets. Participants were included in the study if they were able to provide informed consent and agreed not to take other supplements or medications aimed at preventing URTIs for the duration of the trial (e.g., Echinacea, Vitamin C, zinc, Tamiflu, or Relenza). Exclusion criteria included those with cognitive damage; serious mood disorders or neurological disorders, such as multiple sclerosis; or those with an unstable or serious illness (e.g., renal, hepatic, gastrointestinal, cardiovascular, diabetes, thyroid gland function, malignancy, lung conditions, chronic asthma). Participants were also excluded if they had experienced acute sickness in the previous 2 months; were active smokers or abused nicotine or drugs; had chronic alcohol use (>14 alcoholic drinks per week); were allergic to any of the ingredients in the active or placebo formula; were pregnant or lactating women; were medically prescribed medications that could affect the immune and/or inflammatory response; had participated in a related clinical trial in the 1 month prior; or had treatment for cancer, HIV, or the chronic use of steroids in the past year.

Once enrolled, participants were randomly allocated to one of two groups: either the active (PEA) or placebo group. Randomisation was performed using random allocation site (Sealedenvelope.com, accessed on 10 July 2020) by an individual who was not involved in the trial to ensure both participants and investigators were blinded to the allocation. Those in the PEA (Levagen+^®^) group were required to consume 300 mg of Levagen+ twice daily (morning and evening; 600 mg total daily dose), whereas those in the placebo group were required to consume 300 mg of maltodextrin in the same manner as the active group.

During the study period, participants were asked to complete the SF-8 questionnaire as a measure of health-related quality of life every 4 weeks (baseline, week 4, week 8, and week 12). SF-8 was scored according to Table 1. Upon the completion of the 2 weeks of supplementation, blinded participants were asked to answer an option questionnaire asking what trial product they thought they were on and if they would take it again.

If participants experienced the onset of URTI symptoms (e.g., cough, sneezing, stuffy or runny nose, fever, scratchy or sore throat, or nasal breathing), they were required to record their daily symptoms online, including severity, using the WURSS-24 questionnaire for the duration of the event or up to 2 weeks (whichever occurred first). If the participant’s symptoms continued for more than 2 weeks, they were asked to stop recording the event and seek medical advice. Once symptoms of an event subsided, participants were asked to continue taking the trial product for the remaining duration of the trial period and record any subsequent URTI events.

The primary outcome measure for this study was a change in URTI incidence between groups. Secondary outcome measures included changes in URTI duration, severity (as measured by WURSS-24), general health (as measured by SF-8 questionnaire), number of days off work, and any product tolerance or adverse events.

The sample’s size was calculated using G*power (v3.0.10), accounting for an α probability of 0.05 and powered to 0.95 for a 20% difference in URTI incidence (i.e., 30% vs. 24%); the resulting effect size was 0.74. Group sizes of at least 41 URTI incidents were required; therefore, up to 500 participants were to be recruited with the aim of achieving a minimum of 82 URTI events. Once 82 URTIs were recorded, recruitment into the study was closed, and those enrolled completed the study. Analysis was performed using IBM Statistics (version 25.0 for Windows, IBM, Chicago, IL, USA). Differences between the number of URTIs and symptoms reported per group were assessed using chi-square tests. Changes in URTI symptom severity and duration were analysed using Wilcoxon rank sum (Mann–Whitney U) tests. Statistical significance was set at *p* ≤ 0.05.

## 3. Results

Four hundred and twenty-six participants enrolled in the study, with 398 participants completing full trial requirements. There were 19 withdrawals in the active group and 9 in the placebo group. The Levagen+ group reported four adverse events (diarrhoea, *n* = 3; skin rash, *n* = 1), and the placebo group reported five adverse events (cramps and diarrhoea, *n* = 3; skin irritation, *n* = 1; feeling on edge, *n* = 1). There was no statistical difference between the active and placebo groups for baseline demographics (Table 2).

A total of 87 participants experienced at least one URTI during the study, with a total of 103 URTI episodes recorded. The Levagen+ group reported significantly fewer URTI episodes (39 and 64, respectively; *p* = 0.0056; Table 3) and participants that were sick at least once during the study (32 and 55, respectively; *p* = 0.0116; Table 3) compared to the placebo group.

The number of sick days per episode ranged from 2 to 14 days in both groups, with no significant difference in the median number of sick days between the two groups. For participants reporting a URTI, the Levagen+ group reported a significantly lower severity score for scratchy throat and cough, with hoarseness and the ability to breathe easily trending towards significance (Table 4) when compared to the placebo group.

**Figure 1:**
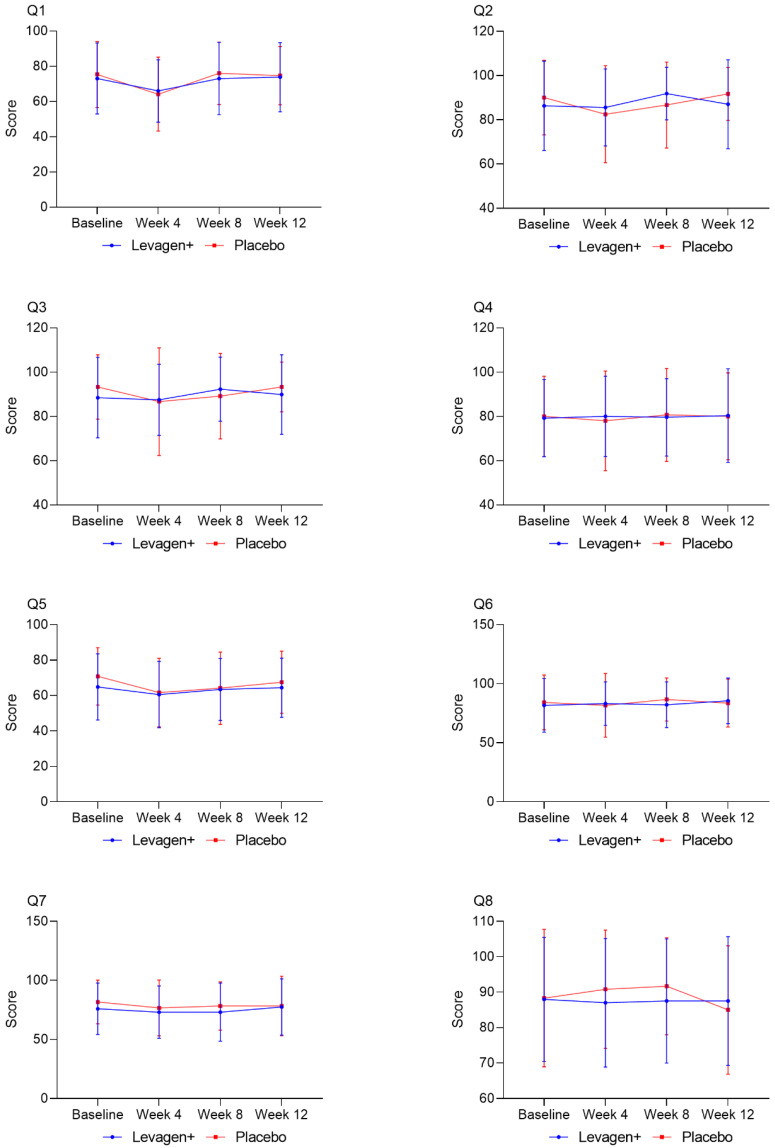
SF-8 outcome measures for each question (Q1 to Q8). There were no significant differences for any question at any collection time point.

Comparisons between groups for the number of people reporting a specific symptom from the WURSS-24 showed no significant difference between groups for any outcome measure (Table 4). No significant differences were observed either within or between groups with respect to the SF-8 general health questionnaire (Figure 1). Overall, compliance for the study was high, with capsule consumption equivalent for both groups (active = 94.5%; placebo = 93.8%).

There was no significant difference between groups for the number of people who thought they were on the active product (83 vs. 92 in the Levagen+ and placebo groups, respectively). Similarly, no significant difference was observed in the number of people willing to take the study product again (80 vs. 91 in the Levagen+ and placebo groups, respectively).

## 4. Discussion

The aim of this study was to assess the effectiveness of PEA (Levagen+) on URTI incidence, duration, and symptom severity in otherwise healthy adults over a 12-week period. Both groups were equally matched, with no between-group differences in participant demographics (Table 2). The primary outcome measure was a change in URTI incidence. Overall, the results showed that the total number of URTI episodes was significantly lower in the Levagen+ group when compared with the placebo group. There was also a significant difference in the severity of scratchy throats and reported coughing between the two groups.

A study by Masek and colleagues (1974) supplemented adults with 600 mg of Levagen+ three times per day (1800 mg of Levagen+ per day) for 12 days. Following supplementation, those with supplementation had fewer episodes of fever and pain and fewer reported headaches and sore throats compared with a placebo group [10]. A second study by Masek and colleagues further showed the prophylactic benefits of PEA in a study conducted on army personnel aged 18 to 20 years old and dosed with 600 mg of PEA three times a day for 3 weeks and then 600 mg once daily for a further 6 weeks. PEA was shown to decrease disease incidence at both weeks 6 and 8 (40% and 32%, respectively) [10]. These data support our findings that Levagen+ supplementation reduces the incidence compared to the placebo group after 12 weeks.

Kahlich and colleagues [19] conducted three similar studies assessing the effectiveness of PEA on influenza symptoms in army personnel over a three-year period, further supporting the results of this study. All three studies showed that those taking PEA had significantly reduced symptoms and were often not diagnosed as flu patients [19]. There were also significant reductions in acute respiratory infections in all three trials for those in PEA groups compared with the placebo (22.7% vs. 34.4%; 19.7% vs. 40.7%; 10.6% vs. 28.8%) [19]. Similarly, the results from the current study found that there were fewer sick days and a reduction in the total number of URTI episodes.

The results of the studies conducted by Masek and colleagues support our findings of a decrease in scratchy throats, sick days, and the total number of URTI episodes in the PEA group. However, the present study did not find a change in reported pain or fever. Based on the mode of action and its anti-inflammatory properties, it would be expected that changes in pain and fever would be observed in the Levagen+ group. Although no significant difference was observed for reported fevers, fewer people in the Levagen+ group reported experiencing a fever. Only 12.8% (5 out of 39) of people in the Levagen+ group who reported an event reported experiencing a fever compared to 25% (16 out of 64) in the placebo group. Fewer people reporting fever may be due to one of two possibilities: Either Levagen+ prevented people from developing a fever or the etiology of illnesses may have been different between groups. As both groups reported an equivalent number of other symptoms (Table 4), including body aches, typically associated with influenza along with fever, it is unlikely that there was much variation in the etiology of the illnesses reported between groups.

There are several other possible reasons for the lack of significance of the reported fever in the present study compared to that of Masek. The first is the difference in dose (1800 mg vs. 600 mg per day). Despite Levagen+^®^ likely having a greater absorption [18] than the PEA used by Masek, the dose used in the present study may have been too low to influence pain and fever. The second reason is the number of people in the study. The present study only reported a total of 21 participants (16 in the placebo and 5 in the Levagen+) reporting fever, and this number may be too low to determine an effect. Another possible reason for the difference in studies could be due to variations in self-reporting or the severity of infections. Participants in the present study may not have become as unwell as those in the Masek study or may have missed reporting the presence of a fever.

One factor potentially affecting the severity of illnesses, and a potential limitation of this study, was that it was conducted during the COVID-19 pandemic. During this period, participants likely experienced fewer URTI events than normal due to isolation, social distancing, and additional sanitation measures being taken. The additional health measures during COVID-19 potentially affected both the number of infections and the severity. People may have been able to rest and recover more during COVID-19 due to both increased isolation and the ability to work from home. The ability to stay home more may have allowed people to rest more and therefore recover faster, limiting the severity of the infection and therefore potentially limiting fever development. However, as all participants were exposed to the same COVID-19 conditions, the results can reasonably be expected to represent the effectiveness of Levagen+, and the only difference due to COVID-19 may be a lower percentage of people experiencing a URTI and symptoms being less severe.

COVID-19 was not a focus of the current study, but it is possible that some of the participants involved experienced COVID-19 while taking the study’s product. The effect that Levagen+^®^ may have on COVID-19 was not determined during this study, but it is feasible that Levagen+^®^ could be effective with respect to treating the symptoms of COVID-19. The reduction in symptoms observed in this study could be a result of a reduction in inflammatory signaling that can also be affected by COVID-19 [15]. Furthermore, in line with the findings of Fonnesu and colleagues and Fessler and colleagues, the reduced number of sick days could also be associated with PEA being able to interfere with viral production [14,15]. A reduction in viral production due to PEA may result in an infection that is unable to replicate and induce symptoms (i.e., reduction in incidence), or the severity of infection may not be as severe.

Another limitation of the study was our inability to collect biological samples. Due to the COVID-19 pandemic, we opted to exclude biological sample collection in order to minimize the potential risk participants might have in traveling to a collection center. Had we been able to collect biological samples, we could have tested for the specific infection the participants had while reporting symptoms. The detection of the infectious agent would have helped classify each reported event into different disease states, enabling us to better understand the effect of Levagen+ in different etiologies. It is possible that PEA may work better in some infections than others. The collection of blood samples would also have allowed us to analyse various pathways that PEA is reported to act upon (e.g., inflammatory cytokines NF-κB and mast cells).

Severity classification is another potential limitation of this study. Due to the number of participants reporting events, grouping events into different severity classifications (e.g., total impact from the WURSS-24 questionnaire) in order to conduct statistical analysis was not possible. It is plausible that PEA may act better on different severities due to its mode of action. For example, PEA has demonstrated the ability to increase β-enzyme activity, which, in turn, enhances the synthesis of the endocannabinoid 2-Arachidonoylglycerol, suggesting that an increased endocannabinoid tone may modulate mast cell degranulation [6,7,20,21]. Corticosteroids have been established to treat moderate or severe symptoms by stimulating endocannabinoid synthesis and signaling [22]. Similarly, Levagen+ modulates endocannabinoid signaling and contributes to the activation of cannabinoid receptors [20,21]. This may mean that those with more severe symptoms may respond better to Levagen+ than those with mild symptoms.

Future studies into the efficacy of PEA would benefit from looking at specific etiologies and severity within URTIs. Further research on the effect of PEA on fever would require larger numbers that focus specifically on the temperature development of participants during an illness. Another study looking specifically at people experiencing COVID-19 or people experiencing Long COVID symptoms would help determine whether PEA is able to be as effective relative to COVID-19 as it is with respect to the common cold and flu.

## 5. Conclusions

The results of this study and those previously published to date support that Levagen+ may be an effective treatment option for the prevention of URTIs and cold and flu symptoms. Overall, Levagen+ was found to be a safe and effective treatment option for those with URTI symptoms, with results indicating that it can decrease the total number of URTI episodes and the symptoms of scratchy throats and coughing.

## Figures and Tables

**Table 1:** SF-8 scoring based on participants’ responses to each question.

Q1	Score	Q2 and Q3	Score	Q4	Score	Q5	Score	Q6 and Q8	Score	Q7	Score
Excellent	100	Not at all	100	None	100	Very much	100	Not at all	100	Not at all	100
Very good	80	Very little	75	Very mild	80	Quite a lot	75	Very little	75	Slightly	75
Good	60	Somewhat	50	Mild	60	some	50	somewhat	50	Moderately	50
Fair	40	Quite a lot	25	Moderate	40	a little	25	Quite a lot	25	Quite a lot	25
Poor	20	Couldn’t do	0	Severe	20	none	0	Could not do daily activities	0	Extremely	0
Very Poor	0	-	-	Very severe	0	-	-	-	-	-	-

**Table 2:** Summary of participants’ baseline demographics.

Parameters	PEA (*n* = 213)	Placebo (*n* = 213)
Gender (*n* male (%))	53 (25)	54 (25)
Age (years)	40.0 ± 12.5	38.9 ± 11.7
Weight (kg)	74.4 ± 18.1	73.2 ± 18.4
Body mass index (kg/m^2^)	26.4 ± 5.5	26.2 ± 6.1

Values are represented as mean ± SD.

**Table 3:** Trial event outcome measures.

	PEA (*n* = 213)	Placebo (*n* = 213)	*p*-Value (Chi-Square)
Number of completed study	194	204	
Number of participants reporting URTI	32	55	0.0116
Total number of URTI episodes reported	39	64	0.0056

**Table 4:** Symptom outcome measures for trial participant data reporting symptoms during a reported event.

Parameter	*n* (%)	PEA Median Severity Score	*n* (%)	Placebo Median Severity Score	*p*-Value (Mann–Whitney U)
Length of episode	39	3	64	4	0.164
Runny nose	32 (82.1)	6	50 (78.1)	6	0.247
Plugged nose	30 (76.9)	5	45 (70.3)	5	0.964
Sneezing	32 (82.1)	4	48 (75.0)	5	0.813
Sore throat	24 (61.5)	5.5	51 (79.7)	6	0.477
Scratchy throat	26 (66.7)	3	46 (71.9)	6.5	0.026 *
Cough	25 (64.1)	2	42 (65.6)	7	0.002 *
Hoarseness	21 (53.8)	3	30 (46.9)	5	0.076
Head congestion	29 (74.4)	5	47 (73.4)	6	0.493
Chest congestion	13 (33.3)	2	29 (45.3)	5	0.333
Feeling tired	33 (84.6)	9	58 (90.6)	9.5	0.861
Headache	23 (59.0)	4	45 (70.3)	6	0.278
Body aches	15 (38.5)	9	33 (51.6)	6	0.533
Fever	5 (12.8)	2	16 (25.0)	2.5	1.000
Total symptoms	36(92.3)	48	64 (100)	49	0.644
Think clearly	35 (89.7)	3	64 (100)	4	0.630
Sleep well	35 (89.7	5	64 (100)	5	0.740
Breathe easily	35 (89.7)	5	64 (100)	3	0.081
Walk, climb stairs, exercise	33 (84.6)	2	64 (100)	1	0.687
Accomplish daily activities	34 (87.2)	2.5	64 (100)	1	1.000
Work outside the home	33 (89.7)	1	64 (100)	0.5	0.704
Work inside the home	33 (89.7)	2	64 (100)	1	0.766
Interact with others	34 (87.2)	1	64 (100)	2	0.957
Live your personal life	33 (89.7)	1	64 (100)	1	0.861
Total impact	35	21	64	20	0.779

Values represented as severity; * significantly different from placebo (*p* < 0.05); % represents the percentage of people in the group reporting symptoms from a reported event.

## Data Availability

The datasets used and/or analysed during the current study are available from the corresponding author upon reasonable request.
